# Usefulness and Potential Pitfalls of Long-Acting Growth Hormone Analogs

**DOI:** 10.3389/fendo.2021.637209

**Published:** 2021-02-24

**Authors:** Kevin C. J. Yuen, Bradley S. Miller, Cesar L. Boguszewski, Andrew R. Hoffman

**Affiliations:** ^1^ Barrow Pituitary Center, Barrow Neurological Institute, Departments of Neuroendocrinology and Neurosurgery, University of Arizona College of Medicine and Creighton School of Medicine, Phoenix, AZ, United States; ^2^ Division of Pediatric Endocrinology, Department of Pediatrics, University of Minnesota, Minneapolis, MN, United States; ^3^ SEMPR, Serviço de Endocrinologia e Metabologia, Departamento de Clínica Médica, Hospital de Clínicas da Universidade Federal do Paraná, Curitiba, Brazil; ^4^ Department of Medicine, VA Palo Alto Health Care System and Stanford University School of Medicine, Palo Alto, CA, United States

**Keywords:** long-acting growth hormone, treatment adherence, growth hormone deficiency, growth hormone replacement, adults, children

## Abstract

Daily recombinant human GH (rhGH) is currently approved for use in children and adults with GH deficiency (GHD) in many countries with relatively few side-effects. Nevertheless, daily injections can be painful and distressing for some patients, often resulting in non-adherence and reduction of treatment outcomes. This has prompted the development of numerous long-acting GH (LAGH) analogs that allow for decreased injection frequency, ranging from weekly, bi-weekly to monthly. These LAGH analogs are attractive as they may theoretically offer increased patient acceptance, tolerability, and therapeutic flexibility. Conversely, there may also be pitfalls to these LAGH analogs, including an unphysiological GH profile and differing molecular structures that pose potential clinical issues in terms of dose initiation, therapeutic monitoring, incidence and duration of side-effects, and long-term safety. Furthermore, fluctuations of peak and trough serum GH and IGF-I levels and variations in therapeutic efficacy may depend on the technology used to prolong GH action. Previous studies of some LAGH analogs have demonstrated non-inferiority compared to daily rhGH in terms of increased growth velocity and improved body composition in children and adults with GHD, respectively, with no significant unanticipated adverse events. Currently, two LAGH analogs are marketed in Asia, one recently approved in the United States, another previously approved but not marketed in Europe, and several others proceeding through various stages of clinical development. Nevertheless, several practical questions still remain, including possible differences in dose initiation between naïve and switch-over patients, methodology of dose adjustment/s, timing of measuring serum IGF-I levels, safety, durability of efficacy and cost-effectiveness. Long-term surveillance of safety and efficacy of LAGH analogs are needed to answer these important questions.

## Introduction

The long-term safety and efficacy of daily recombinant human growth hormone (rhGH) therapy in children with GH deficiency (GHD) are well-studied (1–3). However, maintaining maximal treatment adherence with daily rhGH injections is challenging, not only for children, but also for caregivers and for adults with GHD because of device limitations, pain at injection sites, inconvenience of daily injections, lack of perceived immediate benefits, insurance barriers, and costs (4, 5), leading to frequent dose omissions and treatment cessation (6). Thus, it has been hypothesized that a LAGH analog with a lower injection frequency might help mitigate treatment non-adherence, and thereby potentially improve treatment outcomes.

To this end, many pharmaceutical companies have spent a significant amount of money developing LAGH analogs using a several different yet novel technologies to prolong GH action that may allow for weekly (7–18), bi-weekly (19–21), or monthly administration (22, 23). However, there are theoretical reasons to suspect that LAGH analogs might be inferior to daily rhGH administration. The physiologic secretory pattern of GH occurs in an episodic and pulsatile pattern, with several peaks throughout the day and an increased number in the second half of the night during sleep. Concerns that elevated and non-pulsatile GH exposure may downregulate or desensitize GH receptor signaling were unfounded when a study by Laursen et al. (24) demonstrated that subjects who received continuous subcutaneous infusions of GH over 6 months maintained their serum IGF-I levels within the normal range and did not develop any signs or symptoms of acromegaly. In 1999, the first LAGH analog (Nutropin Depot) was approved and marketed in the United States, but later withdrawn due to manufacturing issues and inferior efficacy reported during post-marketing follow-up (21). The latter problems may be related to increased pain at the injection sites compared to daily rhGH injections. After this experience, the lesson learnt from this experience was that the success of developing future LAGH analogs should not only take into consideration of convenience and ease of injection administration, but also non-inferiority in therapeutic efficacy and a side-effect profile comparable to that of daily rhGH therapy.

## Treatment of GHD in Children and Adults: Where We Were and Where We Are Now

Treatment with rhGH in children with GHD has been well-established for over 35 years in inducing linear growth and attaining adult height appropriate for genetic potential (2). In early studies involving children with GHD, these patients were receiving rhGH that were administered intramuscularly three days a week because this dosage regimen was based on several clinical observations of its effects on growth response (25). The concept of administering subcutaneous rhGH injections daily was first proposed in 1983 by Kastrup et al. (26), and this treatment regimen was found to be efficacious in inducing linear growth and less inconvenient to children. By contrast, current recommendations are weight-based or body surface-based dosing at the start of treatment followed by individualized dosing in accordance with clinical response, with higher rhGH doses preferred for those with greater severity of GHD, and subsequent dose adjustments made according to growth response (1, 2). Serum IGF-I levels are used to monitor for adherence, efficacy and safety (27); if these levels exceed the upper limit of the age-appropriate reference range, then reductions of the rhGH dose should be considered for safety reasons (1, 2). In some cases where treatment response has been inadequate, re-evaluation of other etiologies of short stature and non-adherence are recommended (27).

The first studies assessing the effects of rhGH replacement in adults with GHD was performed in 1989 (28, 29). These studies demonstrated improvements in body composition, exercise capacity, muscle strength, bone remodeling, and dyslipidemia. This prompted a flurry of publications in the literature between 1989 and 1999 in adults with GHD, and the results from many of these studies corroborated with the observations from the two initial pivotal trials in 1989 (28, 29). Further dose-finding clinical studies in various age groups were then performed (30–32) and interestingly, these studies found that adults are inherently more sensitive to the effects of rhGH than children in terms of serum IGF-I generation and rate of side-effects (33), and that males are more responsive to rhGH therapy than females (34, 35). These and other data have resulted in the approval for rhGH replacement in children and adults with GHD by the United States Food and Drug Administration (FDA) and European Medicines Agency in 1985 and 1996, respectively, and translated into several published consensus guidelines for the management of children (2) and adults with GHD (36, 37). Subsequent studies in adults with GHD performed since 1999 have further corroborated the positive effects of rhGH therapy on quality of life, exercise capacity and bone mineral density (38). However, whether rhGH replacement can normalize or decrease the mortality rates in these patients remains debatable. Treatment-related side-effects, mainly related to fluid retention and impaired glucose tolerance, are dose-dependent and related to increased GH sensitivity associated with aging, and are often reversible upon dose reductions or treatment cessation. Caution and close monitoring are recommended with an emphasis on lower rhGH dosing at treatment initiation and maintenance to avoid over-treatment, especially in older, obese, and glucose-intolerant patients as they are generally more susceptible to side-effects. More recent consensus guidelines have emphasized the importance of individualized rhGH dosing in the treatment of adults with GHD, with the primary objective of achieving normalization of age-appropriate serum IGF-I levels (38).

## Long-Acting GH Analogs: Usefulness, Mechanism/s and Current Status

The main indication for the development of LAGH analogs in children and adults with GHD is to improve patient adherence and to ease the burden of chronic daily injections. While many early LAGH analogs were not shown to be effective or practical (39), two LAGH analogs (Eutropin Plus and Jintrolong) are currently being marketed in Asia, one (Somapacitan) was recently approved in the United States, one (Eutropin Plus) was previously approved in Europe but never marketed, and several more close to being considered for regulatory approval in the United States and Europe (**Table 1**). The methodology for creating LAGH analogs can be classified into two broad groups: formulations that create a subcutaneous depot from which native or modified GH is slowly released into the circulation, and formulations that permit rapid absorption from the delivery site in the subcutaneous tissue and delayed removal from the circulation. Other new development methods being studied include reversible complexes to stabilize the GH molecule, fabrication of sustained release preparations that utilize various matrices to bind to the GH molecule, and structural modifications of the GH molecule itself.

**Table 1:** Overview of the development history of LAGH analogs.

Company	LAGH analog	Modification to GH molecule	Frequency of administration	Current status	Research data
**Depot Formulation**		**Depot Chemical**			
Altus Pharmaceuticals	ALTU-238	Long-extended release formulation using protein crystallization technology (22 kDa) (39)	7 days	Althea acquired assets in 2010	No further studies planned
Critical Pharmaceuticals	CP016	Supercritical carbon dioxide, formed when CO_2_ exceeds its thermodynamic critical point, used to create the depot (22 kDa) (39)	14 days	Company under liquidation	Evidence of ongoing studies at other corporations
Genentech	Nutropin Depot^®^	Encapulsated in biocompatible, biodegradable, polylactide-coglycolide polymer microsphere (22 kDa) (40)	14 days	Removed from market (39)	
LG Life Sciences, Ltd	Eutropin Plus™(LB03002)	Microparticles containing GH incorporated into sodium hyaluronate and dispersed in an oil base of medium-chain triglycerides (22 kDa)	7 days	Marketed in Korea for childhood GHD; approved in Europe but not marketed in the EU	Phase 3 trial in CGHD suggest non-inferiority (41), safety data from a Korean registry database in children with growth disorders (42), Phase 2 trial in children with ISS demonstrated non-inferiority and well-tolerated (43)
**PEGylated Formulations**		**PEGylation prolongs *in vivo* mean residence time of GH, through slowing absorption and protection from proteolysis**			
Ambrx	ARX201	30-kDa PEG added to unnatural amino acid incorporated into GH (52 kDa)	7 days	No longer being developed (39) due to PEGylated-containing vacuoles in the epithelial cells of the choroid plexus in monkeys (44)	
Bolder BioTechnology	BBT-031	Site-specific PEGylated GH analog (not available)	7 days (planned)	Preclinical studies (45)	
GeneScience Pharmaceuticals Co, Ltd	Jintrolong^®^	40-kDa PEG attached to GH (62 kDa)	7 days (13, 16)	Marketed in China for CGHD	Phase 3 studies show good IGF-I profile, Phase 4 studies now ongoing
Novo Nordisk	NNC126-0083	43-kDa PEG residue attached to glutamine 141 (65 kDa)	7 days	Unsatisfactory IGF-I profile peak and duration (46)	No longer being developed as of 2011
Pfizer	PHA-794428	Branched 40 kD PEG on N-terminus of GH (62 kDa)	7 days	High rate of lipoatrophy at injection site (47)	No longer being developed as of 2009
**Pro-Drug formulation**		**Mechanism of conversion to active drug**			
Ascendis	TransCon GH^®^(ACP-001)	Unmodified rhGH transiently bound to a PEG carrier molecule *via* a self-cleaving linker that is dependent upon pH and temperature (22 kDa)	7 days (8, 12, 14, 18, 48)	Phase 2 studies in CGHD and AGHD showed comparable GH and IGF-I profile to daily GH dosingPhase 3 studies in CGHD showed positive growth response (49)	Completed Phase 3 study in CGHD and data submitted to FDA and EMAPhase 3 study in AGHD currently planned
**Non-covalent albumin binding GH compound(s)**		**Albumin binding**			
Novo Nordisk A/S	Sogroya^®^(NNC0195-0092)	Single-point mutation in GH, with albumin binding moiety attached (non-covalent albumin-binding properties) (50, 51) (23 kDa)	7 days (52)	Phase 2 studies in CGHD showed comparable IGF-I profile to daily GH dosing (53)Phase 3 studies in AGHD well tolerated (54–56)Approved by the FDA in August 2020 for use in AGHD but not marketed yet	Phase 3 studies in CGHD, Phase 2 studies in SGA
**GH Fusion Proteins**		**Protein fused with GH**			
Ahngook Pharmaceutical Co, Ltd	AG-B1512	Recombinant GH genetically fused to a polypeptide linker and an anti-human serum albumin Fab antibody (~72 kDa)	14 or 28 days (57)	Preclinical studies show IGF-I level elevation sustained for 20 days	Ongoing research
Alteogen	ALT-P1	rhGH fused with NexP™, recombinant a1-antitrypsin (~74 kDa) (58)	unknown	Stopped Phase 2 study in CGHD (59)	
Asterion	ProFuse™ GH	GH binding protein (~82 kDa) (60)	1 month (planned)	Preclinical studies to provide intravascular stores of inactive GH	
Genexine and Handok	GX-H9	rhGH fused to hybrid non-cytolytic immunoglobulin Fc portions of IgD and IgG4 (100 kDa) (61)	7-14 days (62)	Phase 2 studies in AGHD completed (63)Phase 2 studies in CGHD showed reassuring height changes	Phase 3 studies in CGHD with twice-monthly dosing ongoing
Hanmi Pharmaceutical Co	LAPS rhGH(HM10560A)	Homodimeric aglycosylated IgG4 Fc fragment (~51 kDa) (64)	7-14 days (64)	Phase 2 in AGHD show good tolerability	Phase 3 studies in AGHD (65)
JCR Pharmaceuticals	JR-142	Engineered hGH fused at C-terminus with modified human serum albumin at N-terminus (~88 kDa) (66)	7 days	Preclinical trials	Phase 1 study completed (67)
OPKO Health and Pfizer	Somatrogon (MOD-4023)	rhGH fused to three copies of carboxyl-terminal peptide (CTP) of hCG β-subunit (47.5 kDa)	7 days (11, 15)	Phase 2 studies in CGHD (68), Phase 3 studies in AGHD did not meet primary endpoint of truncal fat reduction (17)Phase 3 studies in CGHD showed non-inferior improvement in height velocity with good tolerability	Phase 3 study in CGHD completed (69), and extension studies now ongoing
Teva	Albutropin (TV-1106)	Human serum albumin fused to N-terminus of GH (88 kDa)	7 days (9, 10)	Studies in AGHD discontinued for unknown reason; presumed unfavorable benefit:risk profile	
Versartis	Somavaratan (VRS-317)	Fusion protein of rhGH and the pharmacologically inactive portion of long chains of natural hydrophilic amino acids (XTEN technology)	7, 14 or 28 days (22)	No longer being developed as of 2017 as the Phase 3 study did not meet its primary end-point for non-inferiority comparison against daily rhGH for height velocity in CGHD (22)	

AGHD, adults with GH deficiency; CGHD, children with GH deficiency; EMA, European Medicines Agency; EU; European Union; FDA, Food and Drug Administration; kDa, kilodalton; ISS, idiopathic short stature, PEG, poly(ethylene glycol); rhGH, recombinant human GH; SGS, small for gestational age. Table is modified from Miller BS, et al. (70).

In 1979, after the publication by Lippe et al. (71) using a depot GH preparation in gelatin solution, the next generation of LAGH analogs to be developed was a native rhGH that was micronized, zinc-stabilized and encapsulated in microspheres (Nutropin Depot). Several other LAGH analogs have since been developed and additional studies performed to assess longitudinal growth velocity in children and changes in body composition in adults as primary endpoints (16, 19, 21, 22, 72). Among them, Eutropin Plus, a depot formulation of rhGH, was approved in South Korea in 1992 and in Europe in 2013, but because it was not marketed in Europe for 3 years, its authorization in Europe lapsed. Jintrolong, a PEGylated GH analog, and Somapacitan, an analog of rhGH containing a fatty acid linker which binds reversibly to serum albumin, have been approved for use in children and adults in China and United States, respectively. In September 2017, Versartis, Inc., the manufacturer of Somavaratan (VRS-317), a molecule with extra amino acids added to the head and the tail of GH, reported data from the VELOCITY Phase 3 clinical trial that the drug had failed to meet its primary end-point for non-inferiority when compared against daily rhGH (Genotropin) for height velocity in children with GHD (9.44 cm *vs* 10.70* cm* for those receiving daily rhGH) (22). Based on these findings, the company subsequently made the decision to suspend its manufacture and all clinical trials, and withdrew its United States Investigational Drug Application and equivalent filings in other countries (73). Other LAGH analogs also met the same fate in being discontinued from manufacture for a multitude of reasons: ARX201 due to the development of PEGylated-containing vacuoles in the epithelial cells of the choroid plexus in monkeys (39, 74), NNC126-0083 related to unsatisfactory IGF-I profiles at the doses administered (39, 74), PHA-794428 due to high rates of injection-site lipoatrophy (mainly in women) (47), TV-1106 due to the development of potentially inactivating antibodies (10), and ALTU-238 because the manufacturer had run out of funds (74, 75). On the other hand, studies on the efficacy and safety of other LAGH analogs, such as GX-H9, show promise and are currently being evaluated in ongoing Phase 3 clinical trials (76).

In April 2020, TransCon GH (Lonapegsomatropin^®^, Ascendis Pharma A/S), a sustained-release inactive prodrug of unmodified GH transiently bound to an inert carrier molecule designed to release fully active GH over one week, was granted Orphan Drug Designation by the FDA, after previously receiving Orphan Designation for the treatment of GHD in Europe from the European Commission in October 2019 (https://www.globenewswire.com/news-release/2020/04/15/2016859/0/en/Ascendis-Pharma-A-S-Receives-Orphan-Drug-Designation-for-TransCon-hGH-as-Treatment-for-Growth-Hormone-Deficiency-in-the-United-States.html). In a Phase 1 randomized trial, 44 healthy subjects were treated with 4 different doses of weekly TransCon GH and 2 different doses of daily rhGH. These investigators discovered that TransCon GH was well-tolerated with no binding antibody formation and comparable levels of serum GH and IGF-I were obtained (12). Phase 2 trials in 37 adults with GHD and 53 previously untreated prepubertal children with GHD also revealed comparable safety and efficacy of TransCon GH when compared to daily rhGH (8, 14). In a recent Phase 3 heiGHt trial (NCT02781727) (18) involving 161 previously untreated prepubertal children with GHD that received either weekly TransCon GH or daily Genotropin, annualized height velocity after 1 year was greater with TransCon GH compared to Genotropin (11.2 *vs* 10.3 cm). The preliminary data of the Phase 3 fliGHt trial (NCT03305016) on children with GHD who switched over from daily rhGH injections to once-weekly TransCon injections were presented at the 2020 Endocrine Society Annual Meeting (ENDO 2020) (77). In this study, dose titrations of TransCon GH demonstrated a predictable serum IGF-I response and a similar side-effect profile to daily rhGH therapy. Ascendis Pharma A/S reports that an application to the FDA and European Medicines Agency has been filed based on these data and those from fliGHt and enliGHten (long-term extension) studies with a Prescription Drug User Fee Act date tentatively set for June 25, 2021 (78).

In June 2020, the data from a Phase 3 trial of Somatrogon hGH-CTP (MOD-4023, Pfizer/OPKO Biologics), a long-acting derivative of rhGH modified by the addition of three C-terminal peptide segments from human chorionic gonadotropin to allow for once-weekly delivery, in children with GHD (NCT02968004) were presented at ENDO 2020 (79). Previously untreated prepubertal children with GHD received either weekly Somatrogon hGH-CTP (0.66 mg/kg/wk) or once daily Genotropin (0.24 mg/kg/wk) for 12 months. The annualized height velocity annualized height velocity after 1 year on Somatrogon hGH-CTP therapy was higher and non-inferior compared to Genotropin (10.1 *vs* 9.8 cm). This trial also demonstrated that children receiving Somatrogon hGH-CTP reported good tolerability with lower treatment burden than Genotropin. Based on these data, Pfizer Inc. is expected to file for FDA approval in early 2021 (80).

In August 2020, once-weekly Somapacitan (Sogroya^®^, Novo Nordisk A/S, Denmark) was approved by the FDA for treatment of adult GHD (https://www.fda.gov/drugs/drug-safety-and-availability/fdaapproves-weekly-therapy-adult-growth-hormone-deficiency). Somapacitan is a long-acting human GH derivative to which a small noncovalent albumin-binding moiety is attached to facilitate reversible binding to endogenous albumin, delaying its elimination, and thereby extending its duration of action with little to no accumulation of the drug when administered once-weekly (52). In previous short-term clinical trials, Somapacitan was well-tolerated in healthy adults (81) and in adults and children with GHD (53, 54, 82), and provided similar safety and efficacy to daily rhGH in previously rhGH-treated adults with GHD (54, 55). In a Phase 3, 26-week randomized, controlled multi-center study of 92 adults with GHD treated with Somapacitan or daily Norditropin, Somapacitan was well-tolerated, IGF-I standard deviation scores remained in the therapeutic range, and patients preferred the weekly Somapacitan therapy (54). In another Phase 3 study (REAL 1 trial –NCT02229851) of 257 adults with GHD, Somapacitan treatment for 86 weeks demonstrated superiority to placebo in improving body composition and serum IGF-I levels (56), and was well-tolerated with patients preferring Somapacitan to daily rhGH injections for administration convenience. However, although recently approved by the FDA in the United States, Somapacitan will not be available for commercial use in the foreseeable future as the launch date is yet to be determined by its manufacturer. Conversely, the Phase 3 testing of Somapacitan (REAL 4 trial – NCT03811535) in children with GHD commenced in 2019 and is expected to conclude sometime in 2021 (83).

## Potential Pitfalls of LAGH Analogs

Questions have arisen regarding dosing (particularly whether there are any differences in dose initiation between GH-naïve and switch-over patients from rhGH daily injection and how to adjust dosing during therapy), safety monitoring, and whether LAGH analogs would be as effective and safe compared to daily rhGH because of the differences in pharmacokinetics and pharmacodynamics, as they are not physiologic. Furthermore, because the therapeutic response to daily rhGH injections can be highly variable among patients and may be influenced by multiple factors (e.g., age, time of diagnosis of GHD, gender, body mass index, severity of GHD, quality of life, other pituitary hormone replacements, and GH receptor polymorphisms) (84), it is likely that similar variability to therapeutic responses will be observed with LAGH analogs as well.

It is also anticipated that LAGH analogs will share many, if not all, of the known side-effects of daily rhGH. However, because of the mechanism by which GH action is prolonged and the duration of prolongation, additional safety risks may be present. New safety concerns may include the formation of neutralizing anti-drug antibodies, and growth and metabolic effects related to the altered profile of serum GH and IGF-I levels during therapy. Furthermore, in those drugs where modifications of the GH molecule have been made, there may be a risk of anti-GH antibodies developing. Anti-GH antibodies formed against rhGH given as a daily injection have not been previously shown to be clinically relevant, except in individuals with GH gene deletions (85, 86). If neutralizing antibodies develop against a modified GH molecule, there is a possibility that the individual would not or only partially respond to the unmodified rhGH. As the methods of measuring anti-drug antibodies are not universally consistent, it is important to determine its clinical impact and long-term clinical relevance. Additionally, accurate and reliable anti-drug antibody assays for each LAGH available are required and be made available to clinicians, and when to test for these antibodies while on treatment. Furthermore, it remains unknown if the likelihood of developing anti-drug antibodies is increased if an individual is inevitably switched from one LAGH analog to another.

Another potential pitfall of LAGH analogs is the impact of prolonged elevated serum GH levels after an injection of a LAGH analog resulting in the relative lack of daily GH nocturnal peak and daytime trough profile, unlike the profile with daily rhGH injections at bedtime. This may cause long-term metabolic aberrations since GH is closely involved in the regulation of fat and glucose metabolism, and body composition (39, 87, 88). Furthermore, due to the low levels of GH prior to the next LAGH injection, the use of LAGH in infants and young children with hypoglycemia associated with severe GHD may put them at unnecessary risk. To date, clinical trials of LAGH have not included children less than 2.5 years of age. Growth hormone fusion proteins may have differing therapeutic efficacy profiles because access of the modified GH may be restricted to different key target tissues due to the large overall size of the protein.

The profile of the IGF-I response to each LAGH analog that differs from daily rhGH injections may present with some unique safety concerns. Early epidemiological studies have demonstrated associations of elevated and high normal serum IGF-I levels with increased risk of cancers (89). A specific serum IGF-I cut-off level has not been identified above which there is documented increase in the risk of any known side-effect of daily rhGH injections (1). Depending upon the bioavailability of the LAGH analogs and dose administered, peak serum IGF-I levels achieved with LAGH may need to be relatively higher in order to achieve therapeutic clinical efficacy, but whether there are negative implications of transient elevations of serum IGF-I levels remains to be elucidated. Better understanding of the pharmacokinetic and pharmacodynamic profiles of each individual LAGH analog is required to ascertain the optimal timing of serum IGF-I measurements for both safety and efficacy. Other methodologies of assessing serum IGF-I levels that do not need to take into account of the timing of serum IGF-I measurement in relation to the LAGH analog injections, such as calculating the IGF-I area under the curve, utilizing a mathematical formula and/or measuring other surrogate markers, may be considered but needs further studies to validate their accuracy and reliability. It is also important to avoid inducing supra-physiological IGF-I levels for too long in between LAGH analog injections (39), as this could increase the risk of iatrogenic acromegaly, neoplasia and glucose intolerance. In children with GHD, although monitoring of serum IGF-I levels is recommended, hard evidence supporting this practice or finding a “safe” upper limit to target serum IGF-I levels are lacking (1). Conversely, the question of when to measure serum IGF-I levels does not pose such an issue with daily rhGH injections because these levels stabilize several days after injection, so measurements of that hormone at any time during therapy can been used as a basis to guide dosing. As for LAGH analogs, serum IGF-I levels can rise and fall over several days and may differ in the degree of fluctuations between injections with different LAGH analogs. Therefore, it is still unclear if dosing adjustments of LAGH analogs should be adjusted based on the peak, nadir, or a mathematically calculated mean of several serum IGF-I measurements in between injections, and whether these factors differ between other LAGH analogs.

When new LAGH analogs become commercially available, their use in clinical practice will be determined by coverage through insurance programs or government health policies. In countries with a single payer program, the coverage of LAGH analogs will be assessed not only for safety and efficacy, but also for cost-effectiveness compared with daily rhGH injections. It is possible that insurance carriers and governmental health policies may decide against covering LAGH analogs simply for the sole purpose that LAGH analogs are “convenient” because of the lower frequency of administration, especially if the costs are higher than daily rhGH injections.

Finally, post-marketing surveillance registries are recommended to enable surveillance of LAGH analogs for efficacy, safety, tolerability, cost-effectiveness, and therapeutic durability. Since each individual LAGH analog is unique in its formulation and molecular structure, further studies are needed for each individual LAGH molecule to better understand its pharmacokinetic and pharmacodynamic properties. It would be even more beneficial to set up a combined registry of all LAGH analogs used for treatment of children and adults with GHD in an independent data repository supported by the manufacturers of these compounds. This would enable manufacturers to fulfil their obligatory safety reporting requirements from governmental agencies, facilitate collaborative “real-world” studies, and increase the power of the studies. A global registry would also be an ideal platform to capture the data on the impact of patients being initiated or switched from daily rhGH to LAGH analogs and from one LAGH analog to another.

## Discussion

The major usefulness of LAGH analogs when compared with current rhGH formulations is that the former requires significantly lesser number of injections compared to the latter. However, given the unphysiologic profile of LAGH analogs, new safety concerns have been raised. Prolonged elevated GH levels might induce supra-physiologic serum IGF-I levels and induce iatrogenic acromegaly, neoplasia and glucose intolerance. Nevertheless, these concerns have reassuringly not been substantiated by any robust evidence in numerous published clinical trials thus far. Because each individual LAGH analog has its own unique pharmacokinetic and pharmacodynamic features, safety issues, dose titrations and therapeutic monitoring need to be individually addressed. Pitfalls of LAGH analogs include whether there are pathophysiological long-term implications of prolonged supra-physiologic elevations of serum GH and IGF-I levels, differences in tissue distribution and tissue sensitivity to modified GH molecules, development of anti-drug antibodies, and differences in the side-effect profile compared with daily rhGH injections. The cost-effectiveness of LAGH analogs *vs* daily rhGH injections is another key question that requires resolution. Perhaps the key overarching question is will LAGH analogs increase treatment adherence, and improve treatment efficacy and long-term outcomes without sacrificing patient safety? Though it seems plausible that this presumption might hold true in certain patient populations, this question to date has not been proven and needs to be prospectively tested further in well-designed clinical trials with the answer likely to be dependent on multiple external and individual factors. Clearly there is still much to be learned moving forward in the coming years, but for now, the available data seem to suggest that LAGH analogs are a useful addition to currently available daily rhGH injections, especially for patients who are not coping with the rigors of daily rhGH injections but yet are wanting to continue as they are obtaining clear benefits from this therapy. Finally, we recommend starting surveillance registries once LAGH analogs are approved and become commercially available so that data on efficacy, safety, tolerability, and cost-effectiveness can be collected in large numbers to improve our understanding of the effects of prolonged exposure to these analogs.

## Author Contributions 

All authors listed have made a substantial, direct, and intellectual contribution to the work and approved it for publication.

## Conflict of Interest

KY is an investigator on research grants from Pfizer, Novo Nordisk, and OPKO Biologics, and has consulted for Pfizer, Novo Nordisk, Sandoz, and Ascendis. BM is an investigator on research grants from Alexion, Abbvie, Amgen, Ascendis, Novo Nordisk, OOKO Biologics, Protalix, Sangamo, Sanofi Genzyme, Tolmar, and Takeda and has consulted for Abbvie, Ascendis, BioMarin, Bluebird Bio, Novo Nordisk, Pfizer, Sandoz, Sanofi Genzyme, Tolmar, and Vertice. AH is supported by the Biomedical Research Service of the Department of Veterans Affairs and has consulted for Ascendis, GeneScience, Genexine, Novo Nordisk, Pfizer, and Versartis.

The remaining author declares that the research was conducted in the absence of any commercial or financial relationships that could be construed as a potential conflict of interest.
