# Corrigendum: Usefulness and Potential Pitfalls of Long-Acting Growth Hormone Analogues

**DOI:** 10.3389/fendo.2021.705241

**Published:** 2021-06-28

**Authors:** Kevin C. J. Yuen, Bradley S. Miller, Cesar L. Boguszewski, Andrew R. Hoffman

**Affiliations:** ^1^ Barrow Pituitary Center, Barrow Neurological Institute, Departments of Neuroendocrinology and Neurosurgery, University of Arizona College of Medicine and Creighton School of Medicine, Phoenix, AZ, United States; ^2^ Division of Pediatric Endocrinology, Department of Pediatrics, University of Minnesota, Minneapolis, MN, United States; ^3^ SEMPR, Serviço de Endocrinologia e Metabologia, Departamento de Clínica Médica, Hospital de Clínicas da Universidade Federal do Paraná, Curitiba, Brazil; ^4^ Department of Medicine, VA Palo Alto Health Care System and Stanford University School of Medicine, Palo Alto, CA, United States

**Keywords:** long-acting growth hormone, treatment adherence, growth hormone deficiency, growth hormone replacement, adults, children

## Error in **Table 1**, 5^th^ column, 19^th^ row

In the original article, there was a mistake in **Table 1** as published. In **Table 1**, 5^th^ column, 19^th^ row, the company “Alteogen” under the “Current Status” column was incorrectly stated that the company was “bankrupt in 2009”. This statement is incorrect as the company remains a currently viable bio-tech company globally.

**Table 1:** Overview of the development history of LAGH analogues.

Company	LAGH analog	Modification to GH molecule	Frequency of administration	Current status	Research data
**Depot Formulation**		**Depot Chemical**			
Altus Pharmaceuticals	ALTU-238	Long-extended release formulation using protein crystallization technology (22 kDa) (39)	7 days	Althea acquired assets in 2010	No further studies planned
Critical Pharmaceuticals	CP016	Supercritical carbon dioxide, formed when CO_2_ exceeds its thermodynamic critical point, used to create the depot (22 kDa) (39)	14 days	Company under liquidation	Evidence of ongoing studies at other corporations
Genentech	Nutropin Depot^®^	Encapsulated in biocompatible, biodegradable, polylactide-coglycolide polymer microsphere (22 kDa) (40)	14 days	Removed from market (39)	
LG Life Sciences, Ltd	Eutropin Plus™ (LB03002)	Microparticles containing GH incorporated into sodium hyaluronate and dispersed in an oil base of medium-chain triglycerides (22 kDa)	7 days	Marketed in Korea for childhood GHD; approved in Europe but not marketed in the EU	Phase 3 trial in CGHD suggest non-inferiority (41), safety data from a Korean registry database in children with growth disorders (42), Phase 2 trial in children with ISS demonstrated non-inferiority and well-tolerated (43)
**PEGylated Formulations**		**PEGylation prolongs *in vivo* mean residence time of GH, through slowing absorption and protection from proteolysis**			
Ambrx	ARX201	30-kDa PEG added to unnatural amino acid incorporated into GH (52 kDa)	7 days	No longer being developed (39) due to PEGylated-containing vacuoles in the epithelial cells of the choroid plexus in monkeys (44)	
Bolder BioTechnology	BBT-031	Site-specific PEGylated GH analog (not available)	7 days (planned)	Preclinical studies (45)	
GeneScience Pharmaceuticals Co, Ltd	Jintrolong^®^	40-kDa PEG attached to GH (62 kDa)	7 days (13,16)	Marketed in China for CGHD	Phase 3 studies show good IGF-I profile, Phase 4 studies now ongoing
Novo Nordisk	NNC126-0083	43-kDa PEG residue attached to glutamine 141 (65 kDa)	7 days	Unsatisfactory IGF-I profile peak and duration (46)	No longer being developed as of 2011
Pfizer	PHA-794428	Branched 40 kD PEG on N-terminus of GH (62 kDa)	7 days	High rate of lipoatrophy at injection site (47)	No longer being developed as of 2009
**Pro-Drug formulation**		**Mechanism of conversion to active drug**			
Ascendis	TransCon GH^®^ (ACP-001)	Unmodified rhGH transiently bound to a PEG carrier molecule *via* a self-cleaving linker that is dependent upon pH and temperature (22 kDa)	7 days (8, 12, 14, 18, 48)	Phase 2 studies in CGHD and AGHD showed comparable GH and IGF-I profile to daily GH dosing	Completed Phase 3 study in CGHD and data submitted to FDA and EMA
				Phase 3 studies in CGHD showed positive growth response (49)	Phase 3 study in AGHD currently planned
**Non-covalent albumin binding GH compound(s)**		**Albumin binding**			
Novo Nordisk A/S	Sogroya^®^ (NNC0195-0092)	Single-point mutation in GH, with albumin binding moiety attached (non-covalent albumin-binding properties) (50, 51) (23 kDa)	7 days (52)	Phase 2 studies in CGHD showed comparable IGF-I profile to daily GH dosing (53)	Phase 3 studies in CGHD, Phase 2 studies in SGA
				Phase 3 studies in AGHD well tolerated (54–56)	
				Approved by the FDA in August 2020 for use in AGHD but not marketed yet	
**GH Fusion Proteins**		**Protein fused with GH**			
Ahngook Pharmaceutical Co, Ltd	AG-B1512	Recombinant GH genetically fused to a polypeptide linker and an anti-human serum albumin Fab antibody (~72 kDa)	14 or 28 days (57)	Preclinical studies show IGF-I level elevation sustained for 20 days	Ongoing research
Alteogen	ALT-P1	rhGH fused with NexP™, recombinant a1-antitrypsin (~74 kDa) (58)	unknown	Stopped Phase 2 study in CGHD (59)	
Asterion	ProFuse™ GH	GH binding protein (~82 kDa) (60)	1 month (planned)	Preclinical studies to provide intravascular stores of inactive GH	
Genexine and Handok	GX-H9	rhGH fused to hybrid non-cytolytic immunoglobulin Fc portions of IgD and IgG4 (100 kDa) (61)	7-14 days (62)	Phase 2 studies in AGHD completed (63)	Phase 3 studies in CGHD with twice-monthly dosing ongoing
				Phase 2 studies in CGHD showed reassuring height changes	
Hanmi Pharmaceutical Co	LAPS rhGH (HM10560A)	Homodimeric aglycosylated IgG4 Fc fragment (~51 kDa) (64)	7-14 days (64)	Phase 2 in AGHD show good tolerability	Phase 3 studies in AGHD (65)
JCR Pharmaceuticals	JR-142	Engineered hGH fused at C-terminus with modified human serum albumin at N-terminus (~88 kDa) (66)	7 days	Preclinical trials	Phase 1 study completed (67)
OPKO Health and Pfizer	Somatrogon (MOD-4023)	rhGH fused to three copies of carboxyl-terminal peptide (CTP) of hCG β-subunit (47.5 kDa)	7 days (11, 15)	Phase 2 studies in CGHD (68), Phase 3 studies in AGHD did not meet primary endpoint of truncal fat reduction (17)	Phase 3 study in CGHD completed (69), and extension studies now ongoing
				Phase 3 studies in CGHD showed non-inferior improvement in height velocity with good tolerability	
Teva	Albutropin (TV-1106)	Human serum albumin fused to N-terminus of GH (88 kDa)	7 days (9,10)	Studies in AGHD discontinued for unknown reason; presumed unfavorable benefit:risk profile	
Versartis	Somavaratan (VRS-317)	Fusion protein of rhGH and the pharmacologically inactive portion of long chains of natural hydrophilic amino acids (XTEN technology)	7, 14 or 28 days (22)	No longer being developed as of 2017 as the Phase 3 study did not meet its primary end-point for non-inferiority comparison against daily rhGH for height velocity in CGHD (22)	

AGHD, adults with GH deficiency; CGHD, children with GH deficiency; EMA, European Medicines Agency; EU, European Union, FDA, Food and Drug Administration; kDa, kilodalton; ISS, idiopathic short stature; PEG, poly(ethylene glycol); rhGH, recombinant human GH; SGS, small for gestational age. Table is modified from Miller BS, et al. (70).

The authors apologize for this inadvertent error with the statement and a modified **Table 1** is provided below, where the statement “bankrupt in 2009” is now deleted. This new table does not change the scientific conclusions of the article in any way. The original article has been updated.

